# *Kr-h1*, a Cornerstone Gene in Insect Life History

**DOI:** 10.3389/fphys.2022.905441

**Published:** 2022-04-27

**Authors:** Qianyu He, Yuanxi Zhang

**Affiliations:** ^1^ Heilongjiang Provincial Key Laboratory of Environmental Microbiology and Recycling of Argo-Waste in Cold Region, College of Life Science and Biotechnology, Heilongjiang Bayi Agricultural University, Daqing, China; ^2^ Daqing Municipal Ecology and Environment Bureau, Daqing, China

**Keywords:** Kr-h1, metamorphosis, reproduction, juvenile hormone, ecdysone, behavioral plastisity, neuronal morphogenesis, embryogenesis

## Abstract

Insect life cycle is coordinated by hormones and their downstream effectors. Krüppel homolog1 (Kr-h1) is one of the crucial effectors which mediates the actions of the two critical hormones of insects, the juvenile hormone (JH) and 20-hydroxyecdysone (20E). It is a transcription factor with a DNA-binding motif of eight C_2_H_2_ zinc fingers which is found to be conserved among insect orders. The expression of *Kr-h1* is fluctuant during insect development with high abundance in juvenile instars and lower levels in the final instar and pupal stage, and reappearance in adults, which is governed by the coordination of JH, 20E, and miRNAs. The dynamic expression pattern of *Kr-h1* is closely linked to its function in the entire life of insects. Over the past several years, accumulating studies have advanced our understanding of the role of *Kr-h1* during insect development. It acts as a universal antimetamorphic factor in both hemimetabolous and holometabolous species by directly inhibiting the transcription of 20E signaling genes *Broad-Complex* (*Br-C*) and *Ecdysone induced protein 93F* (*E93*), and steroidogenic enzyme genes involved in ecdysone biosynthesis. Meanwhile, it promotes vitellogenesis and ovarian development in the majority of studied insects. In addition, Kr-h1 regulates insect behavioral plasticity and caste identity, neuronal morphogenesis, maturation of sexual behavior, as well as embryogenesis and metabolic homeostasis. Hence, Kr-h1 acts as a cornerstone regulator in insect life.

## Introduction

Insects represent the largest and most diverse group of animals, with over 70% of all species. They are the first to master flight and establish social societies (Misof et al., 2014). The complex life cycles of insects, which include an abrupt ontogenetic change in an individual’s morphology, physiology, and behavior, undeniably play an important role during insect evolution. These changing life patterns were intimately linked to two hormonal systems, the 20-hydroxyecdysone (20E) and juvenile hormone (JH), which function in both embryonic and postembryonic phases. Generally, pulses of 20E initiate each of the major developmental transitions, while JH is classically viewed as a “status quo” and gonadotropic hormone (Riddiford, 2012; Truman, 2019). 20E induces and directs its functions through the ecdysone receptor (EcR), and a cascade of transcription factors. JH directs 20E action through binding the nuclear receptor methoprene-tolerant (Met) (Jindra et al., 2015) or unidentified membrane receptor (Liu et al., 2015) to control the expression of transcription factor *Krüppel homolog1* (*Kr-h1*) which in turn regulates other stage-specifying genes. However, *Kr-h1* is also regulated by 20E and involved in the 20E-mediated events. Hence, it is a major factor that is critical to life of the insects and that has been repurposed during evolution to regulate many processes. The aim of this review is to summarize the significant advances that have been made in the molecular action of Kr-h1 in insect life during the past few years to get a general understandingof the functions of Kr-h1.

## Conservation of *Kr-h1* Protein in Insects

*Kr-h1* was initially discovered in *Drosophila melanogaster* by characterizing a P-element induced mutant that dies during the prepupal period and fails to complete head eversion. Molecular studies revealed that the *Kr-h1* locus gives rise to two major transcripts (*Kr-h1α* and *Kr-h1β*) which derive from two distinct promoters. The *Kr-h1α* encodes a 791 amino acid protein while *Kr-h1β* has an additional 54 residues at the N-terminus. Both protein isoforms contain a central domain with eight C_2_H_2_ zinc fingers belonging to the Krüppel-like family of transcription factors (Schuh et al., 1986; Pecasse et al., 2000). Subsequently, *Kr-h1* has been cloned in numerous insects from the holometabolan species *Apis mellifera* (Grozinger et al., 2003), *Tribolium castaneum* (Minakuchi et al., 2009), *Aedes aegypti* (Zhu et al., 2010), *Bombyx mori* (Kayukawa et al., 2012)*, Helicoverpa armigera* (Zhang W.-N. et al., 2018), *Bactrocera dorsalis* (Yue et al., 2018), *Sitodiplosis mosellana* (Cheng et al., 2020), *Chilo suppressalis* (Tang et al., 2020), *Colaphellus bowringi* (Guo et al., 2021), and *Harmoniaaxyridis* (Han et al., 2022) to the hemimetabolous species *Blattella germanica* (Lozano and Belles, 2011), *Pyrrhocoris apterus* (Konopova et al., 2011), *Rhodnius prolixus* (Konopova et al., 2011), *Locusta migratoria* (Song et al., 2014), *Nilaparvata lugens* (Li et al., 2018)and *Sogatella furcifera* (Hu et al., 2020). More recently, *Kr-h1* has also been characterized in two most-ancestral insect orders, the Ephemeroptera *Cloeon dipterum* (Kamsoi et al., 2021) and the Odonata *Ischnura senegalensis* and *Pseudothemis zonata* (Okude et al., 2022), which belong to paleopterans. Alignments of the full protein sequences of the orthologs reveal that the eight zinc-finger DNA binding domains are remarkably well conserved among insect orders, as are two additional regions (LPL/PRKR and RXXSVIXXA) at the extreme C-terminus which are proved to be involved in the transcriptional inhibitory activity of *Kr-h1* (Kayukawa et al., 2012; Kayukawa et al., 2016; Li et al., 2018; Cheng et al., 2020). However, the number of amino acids located between the first and second Zn finger, and that between LPL/PRKR and RXXSVIXXA varies with the taxon, especially distinguished from the dipterans. In the dipteran lineage, the sequence between Z1 and Z2 ranges from 34–65 aa, whereas in other taxa, it varies from 9–14 aa. Also, the sequence between LPL/PRKR and RXXSVIXXA is longer in the dipteran lineage (28–60 aa) relative to 14–19 aa in other taxa.

## Expression Profiles of *Kr-h1* in Insects

In *D. melanogaster*, the stage and tissue expression patterns of *DmKr-h1α* and *DmKr-h1β* are distinguished. *DmKr-h1β* is the major transcript in embryogenesis with a broad peak of expression between 8 and 12 h after egg laying and localized to specific neuronal cells (Beck et al., 2004), whereas *DmKr-h1α* predominates throughout larval life. *DmKr-h1α* transiently peaks from the onset of pupariationuntil 6–8 h after pupariation (Pecasse et al., 2000; He et al., 2020), and finally reappears in adults (Pecasse et al., 2000). Unlike *DmKr-h1β, DmKr-h1α* expression spreads to most larval and prepupal tissues, including diploid imaginal discs that are set aside in the larva to later form adult structures (Pecasse et al., 2000; Beck et al., 2005). In *B. mori*, *Kr-h1* also encodes two isoforms (*BmKr-h1α* and *BmKr-h1β*), but the overall expression patterns of these two isoforms are very similar, although the levels of *BmKr-h1β* are far lower than *BmKr-h1α*. The expression of *Kr-h1* is fluctuant during *B. mori* development. Both *BmKr-h1α* and *BmKr-h1β* are continuously expressed in *B. mori epidermis* during the larval stages but disappear completely upon ecdysis to the final larval instar, reappear and peak during the prepupal stage, then disappear again in the pupae. From the late pupal stage, they increase gradually and maintain high levels during the adult stage (Kayukawa et al., 2014).

In other insects, temporal expression profiles of *Kr-h1* mRNA also show abundant levels in larval or nymphal instars, but sudden decrease during the final juvenile stage, particularly in pupae of holometabolan species, such as *T. castaneum* (Minakuchi et al., 2009) and *H. armigera* (Zhang W.-N. et al., 2018), or in the last nymphal instar of hemimetabolous species, such as *B. germanica* (Lozano and Belles, 2011), *P. apterus* (Konopova et al., 2011) and *N. lugens* (Li et al., 2018), and the expression remains undetectable for several days until the adult molt. It is remarkable to note that a transient peak of *Kr-h1* appearsat the prepupal stage of holometabolans or at the mid-end of the penultimate nymphal instar of hemimetabolans respectively, which is of paramount importance that we will discuss in the following section.

## Transcriptional Regulation of *Kr-h1*

*Kr-h1* was initially reported to be a 20E-inducible gene in *D. melanogaster*. Several *Kr-h1* mutants die at the prepupal-pupal transition, or shortly after hatching to a first instar larva. The mutations lead to defects in the timing and level of expression of the key 20E-response genes during metamorphosis or embryogenesis (Pecasse et al., 2000; Beck et al., 2004). Treating salivary glands from wandering larvae or prepupae with 20E increases *Kr-h1* transcript levels by approximately fivefold. This induction seems to be a primary response because it does not require new protein synthesis (Beckstead et al., 2005). Importantly, the regulation effect of 20E on *Kr-h1* expressionhas also been observed in other insects, such as *T. castaneum* (Xu et al., 2018), *H. armigera* (Zhang W.-N. et al., 2018), and *B. mori* (Kayukawa et al., 2014; Zhu Z. et al., 2021). In *B. mori*, the expression patterns of *BmKr-h1α/β* are closely correlated with the titer of ecdysteroids. Treating ovaries or BmN cells of *B. mori* with 20E induced the expression of *BmKr-h1* (Kayukawa et al., 2014; Zhu Z. et al., 2021). Very recently, two 20E *cis*-regulatory regions (CRE) in the *BmKr-h1* promoter had been identified, which also exist in the promoter region of *Kr-h1* genes in other insects, like *D. melanogaster, T. castaneum, A. mellifera, A. aegypti, and A. pisum* (Zhu Z. et al., 2021). A *Kr-h1* regulatory protein named BmKRP had been isolated and demonstrated to be able to bind this CRE. Overexpressing *BmKRP* enhanced the promoter activity of *BmKr-h1*, whereas RNAi knocking-down *BmKRP* decreased the activity. The expression of BmKRP itself was upregulated by 20E through EcR/USP receptor complex interacting with the potential EcRE in *BmKRP* promoter. So the study indicates that 20E stimulates *BmKr-h1* transcription by inducing the expressionof *BmKRP* which then activates *BmKr-h1* by binding the 20E CRE in its promoter (Zhu Z. et al., 2021). However, the regulatory mechanism of 20E-induced *Kr-h1* expression in other insects needs to be confirmed in the future.

**FIGURE 1:**
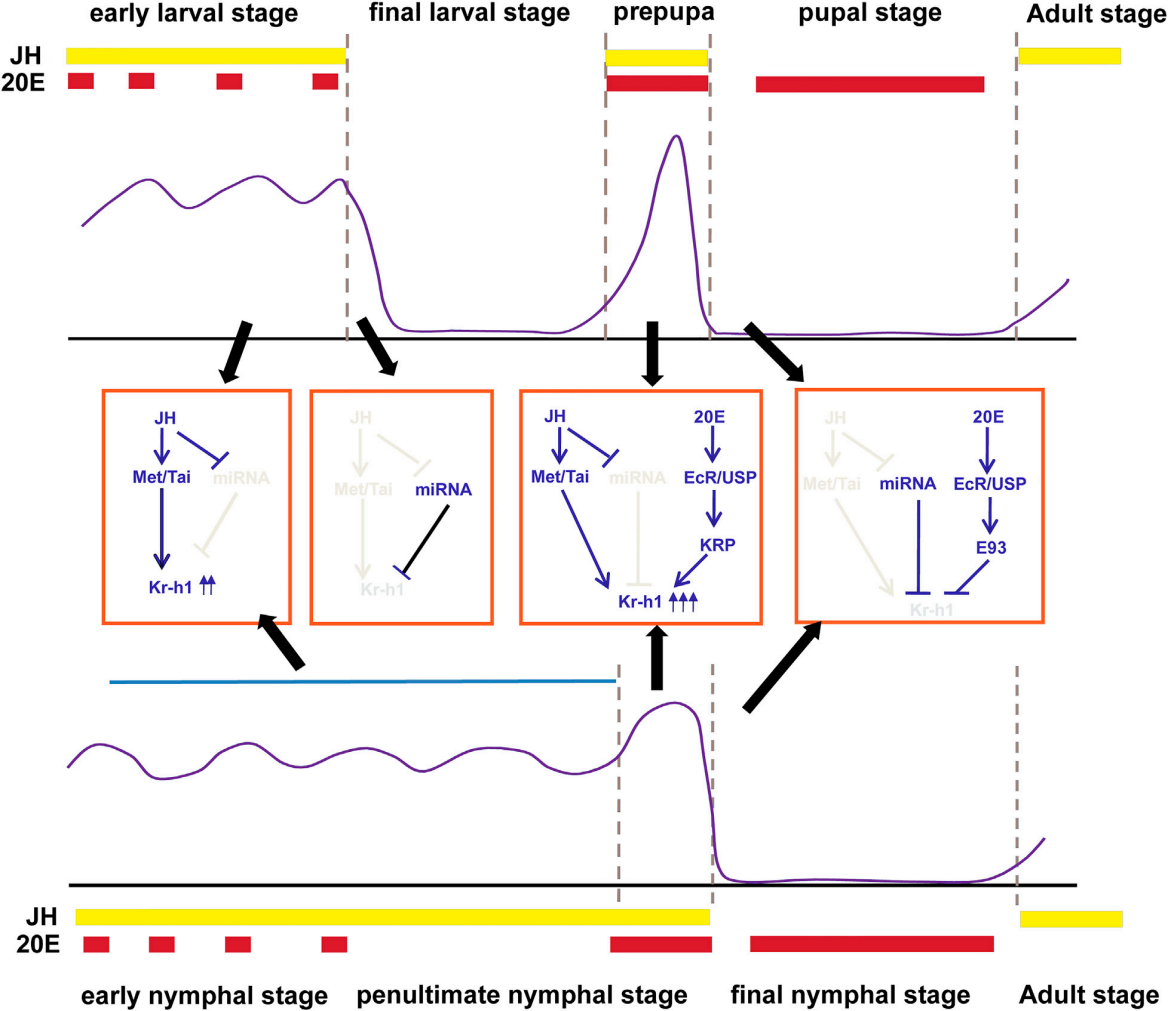
Transcriptional regulation of *Kr-h1* in holometabolous insects (Top panel) and hemimetabolous insects (bottom panel). The expression pattern of *Kr-h1* during insect life history is shown in the purple line. The middle is the regulatory mechanism during each stage of development. The approximate times of 20E pulses and JH levels in holometabolous insects and hemimetabolous insects are shown in red and yellow boxes at the top or bottom of each panel, respectively. Gene expression profiles for *Kr-h1* refers to Belles and Santos (2014) and Martin et al. (2021). JH and 20E titers are from Martin et al. (2021).

It is well known that *Kr-h1* acts as the JH primary response gene in various insect species. The abundance of *Kr-h1* in larval or nymphal stages and the decline of its mRNA levels in pupae or the final nymphal instar coincide well with the JH titer in the hemolymph (Lozano and Belles, 2011; Belles and Santos, 2014; Kayukawa et al., 2014; Jindra, 2019; Truman, 2019; Martin et al., 2021) (Figure 1). Treatment with the JH mimic (JHAM) at the time of onset of metamorphosis or at the end of the penultimate nymphal instar causes rapid and large induction of *Kr-h1* transcripts (Minakuchi et al., 2008b; Minakuchi et al., 2009; Konopova et al., 2011). While suppression of JH biosynthesis or JH signaling in the larval stage or adult leads to a decrease of *Kr-h1* (Minakuchi et al., 2009; Abdou et al., 2011; Konopova et al., 2011; Smykal et al., 2014a; Smykal et al., 2014b; Daimon et al., 2015; Jindra et al., 2015; Nouzova et al., 2021). Moreover, the JH response elements (JHREs) in the *Kr-h1* promoter of several insects have been identified. The JHRE sequences contain a canonical E-box CACGTG or an E-box like motif CACGCG (Kayukawa et al., 2012; Kayukawa et al., 2013; Cui et al., 2014; He et al., 2014), which are the typical binding elements for bHLH-PAS proteins (Kewley et al., 2004). In the presence of JH, the JH receptor Met, belonging to the bHLH-PAS transcriptional family, directly binds to the E box and activates *Kr-h1* transcription in a form of associating with another bHLH-PAS transcription factor known as Taiman/AaFISC/TcSRC (Li et al., 2011; Zhang et al., 2011; Kayukawa et al., 2012), which recently has been demonstrated to be a 1:1 heterodimer (Jindra et al., 2021). This activation initially requires Met to enter the nucleus, which is achieved through interaction with the chaperone heat shock protein Hsp83 and the nucleoporin Nup358 (He et al., 2014; He et al., 2017). JH stimulates the chaperone protein Hsp83 interacting with the bHLH and PAS-B domains of Met in the cytoplasm, and then the association between Hsp83 and the tetratricopeptide repeat (TPR) domain of Nup358 facilitates the nuclear transportation of the entire complex through the nuclear pore (He et al., 2014; He et al., 2017). However, recent studies by Jindra et al. (2021) demonstrated that addition of JHM to the Sf9 cell cultures coexpressed with *T. castaneum* Met and Taiman promoted Met/Taiman dimerization and reduced the interaction between Met and Hsp83. This discrepancy could be attributed to the fact that Jindra et al. (2021) coexpressed Met and Taiman in cells, and the excess Taiman could effectively displace Hsp83 from their overlapping binding sites in the PAS B domain of Met while JH stimulates the interaction of Taiman and Met.

Besides the intracellular signaling, the studies in *A. aegypti* showed that JH could activate *Kr-h1* expression through a hypothetical cell membrane-associated JH receptor *via* PLC signaling. This signaling phosphorylates Met and Taiman by a calcium/calmodulin-dependent protein kinase II (CaMKII), which then positively regulates their binding with JHRE, thus activating the target gene expression (Liu et al., 2015). Therefore, both the cell membrane-based and the intracellular nuclear pathways play important roles in JH regulated *Kr-h1* expression.

In addition to the hormone-receptor regulation mode, JH could induce *Kr-h1* expression through epigenetic modification (Fernandez-Nicolas and Belles, 2017; Roy et al., 2017; Roy and Palli, 2018; George et al., 2019). Studies in *B. germanica* and *T. castaneum* had shown that RNAi depletion of CREB-binding protein (CBP), a protein with acetyltransferase activity (HAT), reduces the JH-driven increase of *Kr-h1* (Fernandez-Nicolas and Belles, 2017; Roy et al., 2017; Roy and Palli, 2018). And Exposure of *T. castaneum* cells to Trichostatin A, a histone deacetylase (HDAC) inhibitor, causes an increase in expression of *Kr-h1* (Roy and Palli, 2018). These suggest that acetylation and deacetylation mediated by CBP and HDACs play an important role in JH induction of *Kr-h1* expression. A recent publication showed that JH suppressed HDAC1, leading to increased levels of histone acetylation and consequently promoting *Kr-h1* transcription (George et al., 2019). In the absence of JH, HDAC1 forms a repressor complex with SIN3 and deacetylates histones that are associated with the *Kr-h1* promoter and then suppress *Kr-h1* expression. When JH is present, the repressor complex is likely replaced by an activator complex which acetylates the core histones at the *Kr-h1* promoter and then facilitates *Kr-h1* expression (George et al., 2019). Recently, this activator is presumed to be CBP (Gaddelapati et al., 2020).

Another factor that needs to be considered regarding Kr-h1 transcriptional regulation is E93. E93 is a helix-turn-helix transcription factor containing a Pip-squeak (Psq) motif, which was first reported to be involved in the regulation of programmed cell death in *D. melanogaster* during the prepupal stage (Baehrecke and Thummel, 1995; Lee et al., 2000). The studies by Urena et al. (2014) found that *E93* mRNA was expressed in the last nymphal instar of *B. germanica* or during the entire pupal period in *T. castaneum* and *D. melanogaster* in a pattern opposite to that of *Kr-h1.* RNAi knockdown of *E93* prevented the decline of *Kr-h1* expression during the final nymphal instar of *B. germanica* or pupal stage of *T. castaneum*, even though the decline of JH titer occurred normally*.* Such observation strongly suggests that *E93* is necessary for the downregulation of *Kr-h1* at the beginning of the pre-adult stage (Urena et al., 2014). Importantly, these findings have been confirmed in other insects, such as *Cimex lectularius* (Gujar and Palli, 2016b), *N. lugens* (Li et al., 2018), and *Gryllus bimaculatus* (Ishimaru et al., 2019).

Besides the transcriptional regulation, microRNA (miRNA) has emerged as a critical post-transcriptional regulator in *Kr-h1* expression*.* It was initially reported in *B. germanica* that miR-2 bound the 3′UTR of *BgKr-h1*mRNA and scavenged *Kr-h1* transcripts during the transition from nymph to adult. Depletion of miR-2 prevents the normal elimination of *Kr-h1*. This study suggests that the abrupt decrease of *Kr-h1* mRNA levels in the final nymphal instar results not only from the decline of JH titer but also from the action of miR-2 (Lozano et al., 2015) (Figure 1). However, this miR-2 binding site in the 3′UTR of *Kr-h1* mRNA was only found to be conserved in hemimetabolous species. Recently, our studies in *D. melanogaster* found that *DmKr-h1* transcription could also be regulated by miRNA. Two miR-927 binding sites were identified in the 3′UTR of *DmKr-h1* mRNA, overexpression of miR-927 decreased the transcriptional levels of *DmKr-h1* and resulted in higher lethality during embryogenesis and metamorphosis (He et al., 2020), which was similar to *DmKr-h1* mutants (Pecasse et al., 2000). Moreover, the expression of miR-927 was found to be repressed by JH, thus forming a positive regulatory loop (He et al., 2020). Similarly, the JH/miRNA/Kr-h1 regulatory axis has also been reported in *L. migratoria*, where let-7 and miR-278 downregulate *LmKr-h1* and JH represses the expression of these two miRNAs (Song et al., 2018). This suggests that the cooperation between hormones and miRNAs ensures the proper regulation of *Kr-h1* expression, and the regulatory axis is conserved during insect evolution.

As stated above, the general expression pattern of *Kr-h1* in hemimetabolous and holometabolous insect development is coordinatedly regulated by the hormones, miRNA, and E93, which could be summarized as follows. 1) in the early juvenile stages, the continuous presence of JH and the low levels of miRNA maintain high expression levels of *Kr-h1*. 2) in the final larval stage of holometabolans, the decrease of JH and the increased expression of miRNA lead to the downregulation of *Kr-h1*. 3) at the prepupal stage or the mid-late stage of the penultimate nymphal instar, a surge of 20E and JH gives rise to the transient peak of *Kr-h1*; 4) upon pupation or ecdysis to the final nymphal instar, gradually decreasing JH and increasing levels of E93 and miRNA lead to the disappearance of *Kr-h1* (Figure 1).

## Kr-h1 Acts as a Repressor of Insect Metamorphosis

It has been well documented that Kr-h1 functions as a transducer of the antimetamorphic action of JH in both hemimetabolous and holometabolous insects. JH precludes insect metamorphosis during the pre-ultimate immature stages, its disappearance in the final instar then permits a metamorphic molt that transforms larvae to adults directly (hemimetaboly) or *via* a pupal stage (holometaboly) (Riddiford, 1994; Belles, 2019; Jindra, 2019; Truman, 2019; Belles, 2020a; Martin et al., 2021). In most insects except for the higher Diptera such as *D. melanogaster* and *A. aegypti* where the “status quo” action of JH is subtle (Liu et al., 2009; Riddiford et al., 2010; Abdou et al., 2011), administration of JH or JHM to the final-instar nymphs, larvae, or pupae leads to repetition of that stage (Riddiford, 1994; Minakuchi et al., 2009; Konopova et al., 2011; Jindra et al., 2013). Conversely, experimental removal of JH at earlier instars causes a precocious metamorphic molt, manifested by heterochronic development of adult characters such as wings and external genitals or formation of miniature pupae in holometabolans (Tan et al., 2005; Minakuchi et al., 2008a; Daimon et al., 2012; Daimon et al., 2015). Regardless of the distinct metamorphic responses to JH among species, exogenous JHM treatment or depletion of JH could increased or decreased the expression of *Kr-h1*, respectively, in the studied insects (Minakuchi et al., 2008a; Minakuchi et al., 2009; Konopova et al., 2011; Smykal et al., 2014b; Daimon et al., 2015), even in *D. melanogaster* and *A. aegypti* (Minakuchi et al., 2008b; Abdou et al., 2011; Nouzova et al., 2021). Moreover, the “status quo” action of JH can be counteracted when the *Kr-h1* expression is blocked. Depletion of *Kr-h1* by RNAi before the JHM treatment at the pupal stage or the final-instar nymphal stage leads to normal adult development instead of repeating the pupal or larval program (Minakuchi et al., 2009; Konopova et al., 2011). Additionally, RNAi knockdown of *Kr-h1* at juvenile stages, such as the fourth instar in *T. castaneum* (Minakuchi et al., 2009), antepenultimate or penultimate instar in *B. germanica* (Lozano and Belles, 2011) or penultimate instar in *P. apterus* (Konopova et al., 2011; Smykal et al., 2014b) induces precocious pupation or premature adult development. Meanwhile, as mentioned above, a transient peak of *Kr-h1* at the prepupal stage of holometabolous insects, which may be regulated by a combination of JH and 20E, has been demonstrated to be essential for the formation of the pupa (Urena et al., 2016). Depletion of this specific *Kr-h1* peak triggers a direct transformation of the larva into adult form, bypassing the pupal stage (Urena et al., 2016). On the other hand, studies in *B. mori* showed that constitutive overexpression of *Kr-h1* caused prepupal arrest with unusually thin-skinned cocoon (Kayukawa et al., 2014). In *D. melanogaster*, ectopic expression of *Kr-h1* in the histoblast during metamorphosis blocks normal differentiation of the abdominal *epidermis* and causes missing or short dorsal midline bristles in the adult, a phenotype that resembles the effect of exogenous JH treatment (Minakuchi et al., 2008b). In summary, all these data demonstrate that Kr-h1 functions as a universal JH-dependent repressor of metamorphosis in insects undergoing both holometaboly and hemimetaboly.

**FIGURE 2:**
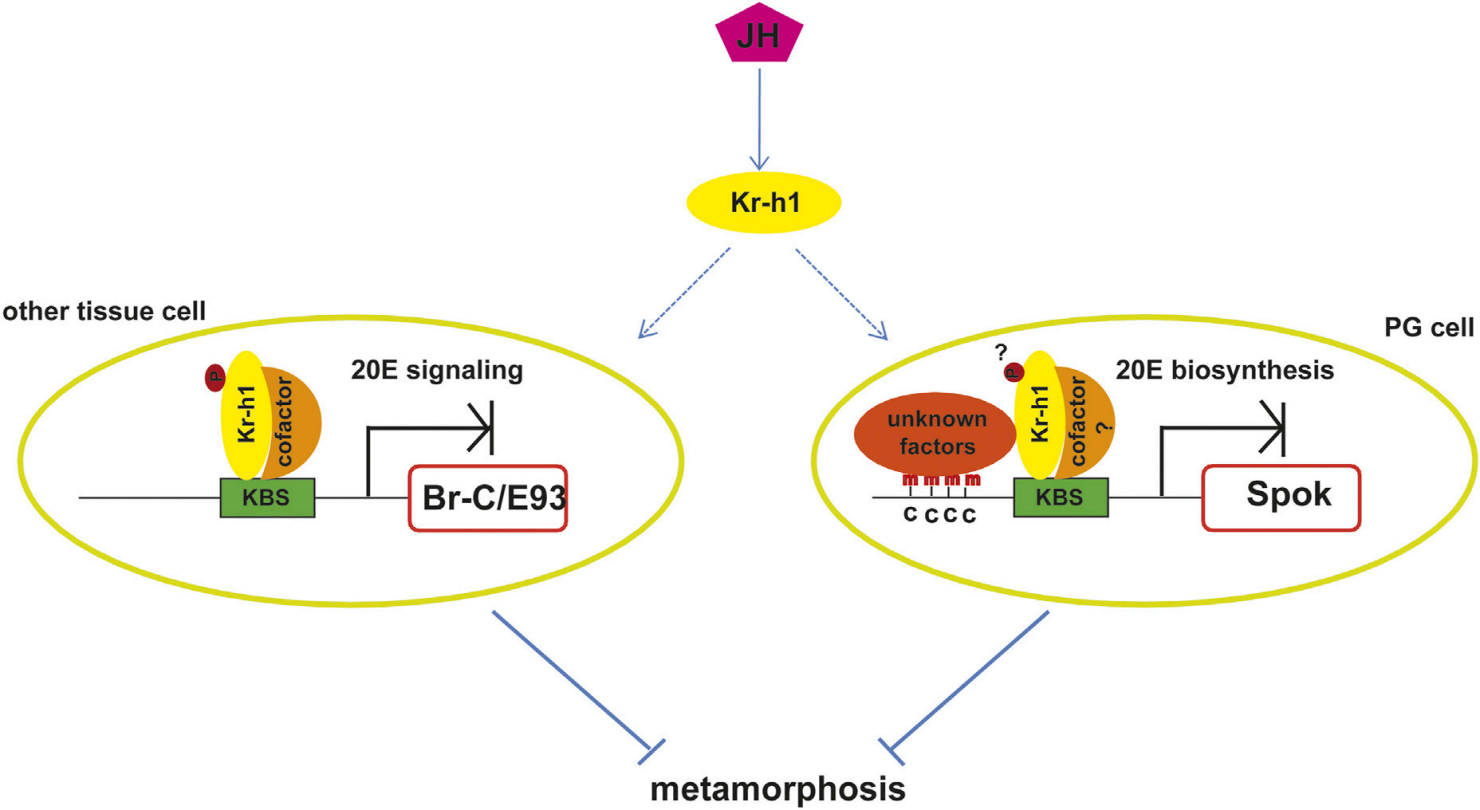
Molecular mechanisms of the anti-metamorphic action of Kr-h1. JH prevents insect metamorphosis by activating *Kr-h1* expression. Kr-h1 binds directly to the KBS in the promoters of pupal specifier gene *Br-C* or adult specifier gene *E93* to inhibit their expression, which in turn prevent pupal metamorphosis or adult metamorphosis. This action requires the phosphorylation of Kr-h1 and the recruitment of cofactors. Kr-h1 could also prevent metamorphosis *via* repressing the expression of enzymes involved in 20E biosynthesis, such as *Spok*. The binding of Kr-h1 to the KBS in the promoter of *Spok* induces the adjacent DNA methylation by unknown factors, which leads to the repression of *Spok* expression. Whether this action depends on the phosphorylation of Kr-h1and the association with cofactors is unknown. KBS, Kr-h1 binding site; p, phosphorylation modification; c, cytosine; PG, prothoracic gland.

Regarding the molecular mechanism by which Kr-h1 mediates the anti-metamorphic effect of JH, various results show that it mainly depends on the direct repression of the expression of the pupal specifier gene *Broad-Complex* (*Br-C*) and the adult specifier gene *Ecdysone induced protein 93F* (*E93*) (Figure 2). The role of *Br-C* as the pupal specifier has been extensively confirmed in the holometabolan species. For example, in *D. melanogaster*, *Br-C* null mutation results in death at the time of pupariation (Kiss et al., 1976; Kiss et al., 1988), and in other insects RNAi depletion of *Br-C* results in the failure of larvae to undergo normal pupal transition or a mix feature of larva, pupa and adult (Zhou and Riddiford, 2002; Uhlirova et al., 2003; Konopova and Jindra, 2008; Parthasarathy et al., 2008; Suzuki et al., 2008; Ding et al., 2020). JH prevents pupation by restricting *Br-C* expression in young larvae until the onset of the final larval instar when the decline of JH allows the induction of *Br-C* by 20E, which then triggers the pupal formation. Removal of the JH-producing corpora allata glands causes ectopic *Br-C* induction as well as precocious pupation, whereas exposure of larvae to JH prevents both *Br-C* transcription and pupation (Zhou et al., 1998; Reza et al., 2004). Likewise, *Kr-h1* mutations result in precocious *Br-C* expression, and the precocious expression could not be suppressed by exogenous JHM (Huang et al., 2011). This indicates that *Kr-h1* mediates the JH-inhibited precocious *Br-C* expression during larval stages.

The conception that *E93* functions as a universal adult specifier in both hemimetabolous and holometabolous insects is afforded by the research of Urena *et al.* The authors found that *E93* is highly upregulated during the final nymphal instar or the entire pupal period. RNAi depletion of *E93* in the penultimate nymphs of *B. germanica*or mid final instar larvaeof *T. castaneum* prevents the nymphal-adult or pupal-adult metamorphic transition, inducing multiple reiterations of nymphal molts or producing a supernumerary second pupa, even in the absence of JH (Urena et al., 2014). At the same time, Belles and Santos (2014) found that *Kr-h1* repressed *E93* expression in *B. germanica*, and later, Urena et al. (2016), confirmed the same interaction in *T. castaneum*, thus indicating that repression of *Kr-h1* upon *E93* prevented the precocious adult metamorphosis. In the same way, Urena et al. (2016) used RNAi experiment to deplete the transient peak of *Kr-h1* expression at the prepupal stage in *T. castaneum* and found that this led to a precocious upregulation of *E93* and hence to a direct transformation of the larva into the adult, bypassing the pupal stage (Urena et al., 2016). Thus, the MEKRE93 pathway which comprises Met, Kr-h1, and E93 is the essential axis regulating adult morphogenesis (Belles and Santos, 2014; Belles, 2020b). Subsequent research in Shinoda lab elucidated that *Kr-h1* mediated *Br-C* and *E93* transcriptional suppression *via* directly binding to the Kr-h1 binding sites (KBS, TGACCTNNNNYAAC) in their promoters (Figure 2). When KBS was deleted from the promoter, the repression was abolished (Kayukawa et al., 2016; Kayukawa et al., 2017). Thus *Kr-h1* prevents precocious metamorphosis of juvenile insects to adults by directly repressing the transcriptional expression of *Br-C* and *E93*.

Remarkably, it is paradoxical that the repression of Kr-h1 on *Br-C* in larvae seems not to operate during the larva-pupa transition as the pulse of *Br-C* parallels that of *Kr-h1,* and it is strongly suppressed after *Kr-h1* RNAi, as shown in *T. castaneum* (Urena et al., 2016). Consistently, overexpression of *Kr-h1* in *B. mori* during this stage does not prevent the *Br-C* pulse (Kayukawa et al., 2014). Furthermore, exogenous JHM treatment in the prepupae of *D. melanogaster* and *T. castaneum* induces both *Kr-h1* and *Br-C* expression (Minakuchi et al., 2008b; Minakuchi et al., 2009). One explanation is that the pulse of *Kr-h1* during the prepupal stage inhibits the upregulation of *E93*, which is also a strong repressor of *Br-C*. So the induction effect of Kr-h1 on *Br-C* during the prepupal stage may result from the repression of *E93*. This explanation seems to be plausible. However, the expression of *E93* is always downregulated in juvenile stages. Thus according to this explanation, *Kr-h1* should also stimulate *Br-C* expression in early larval stages, which is obviously not the case. Another possibility might be that the stage-specific regulatory effect of Kr-h1 on *Br-C* results from differences in the cell-autonomous factors at each stage, such as different cofactors (co-repressor/co-activator), epigenetic modification of the promoter, or post-translational modification of Kr-h1. A study in mosquitoes showed that Kr-h1 acts either as a transcriptional activator or a repressor in female reproduction through directly binding to the regulatory regions of its target genes in a promoter-specific manner (Ojani et al., 2018). Recently, Wu et al. (2021) demonstrated that Kr-h1 recruited different cofactors to function as activators and repressors at distinct developmental stages. During the juvenile stage, Kr-h1 recruits a corepressor called C-terminal binding protein (CtBP) to repress the transcription of *E93* and prevent adult formation. While in the adult stage, Kr-h1 recruits the coactivator CBP to promote vitellogenesis, and the phosphorylation status of Kr-h1 is indispensable for the cofactors recruitment (Figure 2). Nevertheless, more research is still needed to gain a unified understanding of the stage-specific regulatory effect of *Kr-h1* on *Br-C.*

Besides antagonizing ecdysone action through decreasing the expression of *Br-C* and *E93* to mediate the anti-metamorphic action of JH, *Kr-h1* could also inhibit ecdysone biosynthesis in the prothoracic gland by directly suppressing the expression of steroidogenic enzyme genes (Zhang T. et al., 2018; Liu et al., 2018). Kr-h1 binds to the KBSs in the promoter of *Spok* which encodes a rate-limiting enzyme involved in ecdysteriodogenesis and then induces DNA methylation at cytosines around the KBS. This action in turn inhibits transcription of *Spok* and negatively regulates ecdysone biosynthesis (Figure 2). Hence, *Kr-h1* transduces the anti-metamorphic action of JH through repressing both 20E signaling and 20E biosynthesis (Figure 2).

## Kr-h1 Acts as an Activator in Female Reproduction

In addition to repressing metamorphosis, *Kr-h1* has also been found to function in female reproduction. In most insects, vitellogenesis is the central event of female reproduction, which involves the production and secretion of vitellogenin (Vg) by the fat body, followed by transportation of Vg through the intercellular spaces in the follicular epithelium (known as patency) and subsequent uptake by maturing oocytes *via* Vg receptor-mediated endocytosis (Valle, 1993; Roy et al., 2018; Santos et al., 2019; Wu et al., 2020). It has been well established that insect female reproduction is coordinately regulated by JH, 20E, nutrients, and miRNA (Roy et al., 2018; Wu et al., 2020). In hemimetabolous and basal holometabolous insects, JH is the main hormone that stimulates vitellogenesis, while 20E takes over the role of the leading hormonal regulator among the insect orders of Diptera, some hymenopterans, and lepidopterans (Raikhel and Dhadialla, 1992; Valle, 1993; Wyatt and Davey, 1996; Roy et al., 2018; Santos et al., 2019). As mentioned above, *Kr-h1* is regulated by both JH and 20E. Hence, *Kr-h1* is also involved in female reproduction. However, the role of *Kr-h1* in insect vitellogenesis varies across species. In *L. migratoria*, *Kr-h1* RNAi prevents JH-induced *Vg* expression in the fat body along with blocked oocyte maturation and arrested ovarian development. Meanwhile, the JH-induced intercellular space in the follicular epithelium fails to occur after *Kr-h1* depletion. *Kr-h1* RNAi blocks lipid accumulation in the primary oocytes (Song et al., 2014). Similarly, *Kr-h1* knockdown leads to reduction of *Vg* transcript levels and impaired oocyte maturation in *H. armigera* (Zhang W.-N. et al., 2018), *B. dorsalis* (Yue et al., 2018), *C. suppressalis* (Tang et al., 2020), *S. furcifera* (Hu et al., 2020), *C. bowringi* (Guo et al., 2021) and *H. axyridis* (Han et al., 2022). However, in the hemipteran linden bug *P. apterus*, RNAi knockdown of *Kr-h1* neither reduced *Vg* expression nor affected ovarian development, whereas JH deprivation or knockdown of *Met* or *Tai* did (Smykal et al., 2014a). Likewise, in the common bed bug *C. lectularius*, knockdown of *Kr-h1* did not affect Vg synthesis and fecundity (Gujar and Palli, 2016a). This suggests that in the true bugs (Heteroptera) other transcriptional factors might mediate JH-regulated vitellogenesis. Studies in the coleopteran *T. castaneum* showed that RNAi depletion of *Kr-h1* only resulted in about 30% reduction in *Vg* mRNA levels while knocking down the expression of *JHAMT*, a key enzyme involved in JH biosynthesis, or *Met* caused about 80–90% reduction (Parthasarathy et al., 2010). Later studies demonstrated that JH functions through an insulin-like peptide (ILP) signaling pathway to stimulate Vg production (Sheng et al., 2011). Application of JH to previtellogenic female beetles induced the expression of ILP2 and ILP3 which acts through the insulin-like peptide signaling pathway to phosphorylate FOXO, leading to its export from the nucleus, thus triggering the expression of *Vg*. Knockdown of *JHAMT* or *Met* decreased ILP expression and induced FOXO nuclear localization, resulting in the binding of FOXO to the FOXO response element within the *Vg* promoter, thus repressing Vg synthesis (Sheng et al., 2011). This research indicates that in *T. castaneum*, JH stimulates Vg synthesis through inducing ILP expression and activating the ILP signaling pathway, but whether this mechanism is conserved among insects where vitellogenesis is regulated by JH remains to be confirmed. The role of *Kr-h1* in this regulatory pathway remains unclear.

In the dipterans such as *D. melanogaster* and *Ae. aegypti*, *Vg* expression is mainly induced by 20E (Bownes, 1982; Valle, 1993). In *Ae. aegypti*, Vg synthesis in the fat body is stimulated by a blood meal which triggers the release of the ovarian ecdysteroidogenic hormone (OEH) from the medial neurosecretory cells of the brain. The OEH activates follicular cells of the ovary to produce ecdysone, which is converted into the active 20E in the fat body. 20E then stimulates Vg expression *via* the ecdysone pathway. The 5′ upstream regulatory region of the mosquito *Vg* contains several binding sites for EcR-USP and the early and early-late 20E-inducible genes, such as E74B, E75A, Br-C-Z2, βFTZ-F1, and HR3, which indicates a combinatorial control of *Vg* expression by 20E (Kokoza et al., 2001; Martin et al., 2001; Chen et al., 2004; Sun et al., 2004; Zhu et al., 2006; Cruz et al., 2012; Mane-Padros et al., 2012). The role of JH in mosquito reproduction is to make the fat body competent for Vg synthesis during the previtellogenic stage. The translation of the competence factor βFTZ-F1 that potentiates the activation of 20E-responsive genes is controlled by JH (Zhu et al., 2003; Zhu et al., 2006). Neither blood meal nor 20E treatment can induce the expression of *Vg* when *βFTZ-F1* is depleted (Zhu et al., 2003). Besides, JH acts *via* Met to activate ribosomal biogenesis in the fat body to facilitate the massive production of Vg (Wang et al., 2017). Kr-h1 functions as a transcriptional activator or a repressor in JH/Met-regulated female mosquito reproduction through directly binding to the regulatory regions of target genes (Ojani et al., 2018). RNAi depletion of *Kr-h1* remarkably reduces the length of primary follicles and egg production after blood feeding (Ojani et al., 2018; Saha et al., 2019). Additionally, Kr-h1 could act synergistically with Hairy in the repression of JH/Met target genes during previtellogenic development. Knockdown of *Kr-h1* and *Hairy* resulted in overlapping changes at the transcriptomes level. Only a simultaneous knockdown of *Kr-h1* and *Hairy* could fully phenocopy the effect of *Met* RNAi (Saha et al., 2019). Recently, in the brown planthopper *N. lugens*, it has been demonstrated that the N-terminal zinc-finger domains of Kr-h1 interact directly with Hairy in modulating gene transcription (Mao et al., 2020).

In *D. melanogaster*, it has been well documented that 20E is responsible for oogenesis and the high rate of Vg synthesis in the fat body and JH is essential for the uptake of Vg into oocytes from the hemolymph (Bownes, 1982; Soller et al., 1999). However, JH signaling is also involved in oogenesis. The decreased egg-laying rate was observed in *Met* or *Gce* null mutants and JH deficient flies (Abdou et al., 2011), which later was found to be caused by the accumulation of mature eggs in the ovary (Luo et al., 2021). Using the *JHRR-LacZ* which recapitulates the responsiveness of *Kr-h1* to JH and Met/Gce as an activity indicator of JH signaling (He et al., 2014), Luo et al. (2021) found that JH signaling was activated in ovarian muscle cells and adult fat body. RNAi knockdown of *Met/Gce* or *Kr-h1* in ovaries or adult fat body significantly decreased fecundity, with reduced oviposition, increased ovary size, and accumulation of mature eggs in the ovary, along with reduced egg length. Meanwhile, the expression levels of *laminin* and *collagen IV* that participate in the assembly of ovarian muscle extracellular matrix (ECM) were strongly decreased in the JH signaling-deficient flies. The ECM components are indispensable for ovarian muscle contraction that eventually generates a mechanical force. Therefore, JH signaling promotes ovulation and maintains egg shape by inducing the expression of ECM genes (Luo et al., 2021). Besides, the authors demonstrated that JH signaling could directly regulate germline stem cells (GSCs) maintenance in the ovary. RNAi knockdown of *Met* and *Gce* or *Kr-h1* targeted to the cap cells of the ovary led to significantly declined numbers of GSCs (Luo et al., 2020). These experiments indicate that the JH/Met/Kr-h1 signaling also functions in *D. melanogaster* oogenesis.

In lepidopterans, hormone control of reproduction appears to have evolved independently in different lineages. For example, in *H. armigera* and *Manduca sexta*, which synthesize Vg in adults, JH plays a significant role in regulating female reproduction, whereas in *B. mori*, where vitellogenesis proceeds before adult emergence in the absence of JH, 20E is the essential hormone controlling egg development (Roy et al., 2018; Khalid et al., 2021). Recent findings in *B. mori* showed that *Kr-h1* was highly expressed in ovaries during the late pupal and adult stages and the expression was induced by 20E (Zhu Z. et al., 2021). RNAi-mediated depletion of *Kr-h1* in female pupae during the late pupal stage resulted in abnormal oocytes with less yolk protein deposition and partially transparent chorion. Kr-h1 exerts this function through upregulating the expression of the Vg receptor which affects the yolk protein absorption process, or probably *via* activating the transcription factorAp-1and FOXG1 which then regulate genes involved in the metabolic-related pathways and the nutrient absorption process to modulate the oocyte maturation (Zhu Z. et al., 2021; Zhu Z. D. et al., 2021).

In summary, among insect orders, the female reproduction is coregulated by a complex interaction between JH, 20E, and nutritional signaling pathways, and *Kr-h1* plays crucial roles among these regulatory pathways.

It is interesting to note that Kr-h1 functions as a transcriptional repressor in insect metamorphosis and switches as an activator in adult reproduction. As mentioned previously, the studies by Wu *et al.*(2021)demonstrated that the dual functions of Kr-h1 depend on the property of the recruited cofactors. The high expression levels of the corepressor CtBP and coactivator CBP in the juvenile stage and adult stage, respectively, might explain the distinct Kr-h1 complexes forming in these two stages. However, whether other factors are involved in stimulating the specific complexes remains to be investigated.

## Other Biological Functions of *Kr-h1* in Insect Life

In addition to the roles in insect metamorphosis and reproduction, *Kr-h1* is also necessary for many other aspects of insect development, such as neuron fate specification and morphogenesis (Shi et al., 2007; Hewes, 2008; Fichelson et al., 2012; Marchetti and Tavosanis, 2019), social hierarchy and behavioral plasticity (Whitfield et al., 2003; Grozinger and Robinson, 2007; Shpigler et al., 2010; Gospocic et al., 2021), maturation of sexual behavior (Duportets et al., 2012; Gassias et al., 2021), embryogenesis (Fernandez-Nicolas and Belles, 2017) and metabolic homeostasis (Kang et al., 2017). Most of these physiological processes are also regulated by JH and 20E signaling.

### Neuronal Morphogenesis

In *D. melanogaster*, *Kr-h1* is downregulated in mushroom body (MB) neurons both at the time of initial morphological differentiation and during metamorphic neurite remodeling. Overexpression of *Kr-h1* in developing MB neurons inhibits axon remodeling and blocks MB neuron morphogenesis (Shi et al., 2007)*.* During development, four major types of MB neurons (γ, α'β', pioneer αβ and αβ) are sequentially produced, with γ, α'β' neurons arising during early larval stages or late larval stages respectively, and pioneer αβ and αβ are generated during early metamorphosis (Lee et al., 1999). TGF-β signaling promotes the expression of EcR-B1, which is required in MB neuronal progenitors to consolidate the α'β' fate. *Kr-h1* antagonizes the effect of TGF-β signaling, leading to decreased levels of EcR-B1 and subsequently a significant reduction in the number of α'β' neurons. This suggests that Kr-h1 negatively modulates the 20E signaling response to maintain the correct MB neuronal identity (Marchetti and Tavosanis, 2019). Similarly, during *D. melanogaster* photoreceptor maturation, transient repression of *Kr-h1* by the conserved homeobox protein Orthodenticle (Otd) and EcR is required to enable neuronal maturation to proceed normally (Fichelson et al., 2012). Thus, *Kr-h*1 appears to exhibit an antimorphogenetic activity in developing and remodeling neurons. However, in the honey bee, the increased expression levels of *Kr-h1* accompany the neurite outgrowth and synapse formation in the MB (Grozinger et al., 2003; Whitfield et al., 2003), which indicates that Kr-h1 acts as a positive regulator in honey bee neurite outgrowth.

### Behavioral Plasticity And Caste Identity

Behavioral plasticity is key to animal survival. Social insects provide an excellent model system for this study. The adult honey bee possesses a remarkable trait that is the behavioral transition from inhive tasks to outside tasks. Upon emergence, worker honey bees begin the task of “nursing” behavior, where they are responsible for the feeding and care of the queen and brood. Approximately 1 week later, they change to new roles, such as storing and processing food; when they are 3 weeks of age, worker bees begin to forage fornectar, pollenor water outside of the hive (Robinson, 1992). Increased levels of *Kr-h1* in the brain are associated with the behavior transition from nursing to foraging. The expression levels of *Kr-h1* are found to be significantly higher in foragers than in nurses and all other behavioral groups (Whitfield et al., 2003; Grozinger and Robinson, 2007). The factors that regulate *Kr-h1* expression, such as age, JH, and brain octopamine which could accelerate the transition to foraging, have been extensively examined. But the results show that *Kr-h1* expression is not regulated by any of these factors, although JH reduces the effect of queen mandibular pheromone on decreasing *Kr-h1* expression (Grozinger et al., 2003; Grozinger and Robinson, 2007). Later, using behavioral manipulations and pharmacological treatments, Fussnecker and Grozinger demonstrated that high *Kr-h1* levels in foragers resulted from the cGMP-mediated physiological changes in the brain that occurred early in the transition, and the *Kr-h1* promoter contained a conserved potential cGMP response element (Fussnecker and Grozinger, 2008). However, in contrast to the honey bee, bumble bee foragers do not have higher *Kr-h1* levels relative to nurses. Instead, in bumble bees, *Kr-h1* is associated with JH-mediated division of worker reproduction and dominance rank (Shpigler et al., 2010; Pandey and Bloch, 2021). Bumble bees are primitively eusocial insects that there are no obvious morphological differences between the queen and the workers. Direct contact with the queen is required to inhibit worker reproduction (Breed, 1976; Brown et al., 2003). However, JH treatment of workers causes a dose-dependent increase in oocyte length, even in the presence of the inhibitory queen, but does not affect worker task. High levels of JH are associated with worker ovary activation and reproduction, and high dominance rank in queenless workers. An individual with reduced JH levels is less likely to acquire a high dominance rank in queenless workers, and this effect is fully reverted by topical treatment with JH (Bloch et al., 2000; Pandey et al., 2020). Thus, in bumble bee, JH influences reproduction as well as other behaviors related to dominance. *Kr-h1* levels are found to be higher in dominant workers with active ovaries relative to subordinated individuals with undeveloped ovaries in queenless groups (Shpigler et al., 2010). Knockdown *Kr-h1* expression in bumble bees by a perfluorocarbon nanoparticles-based RNA interference protocol showed that bees with reduced *Kr-h1* transcript abundance exhibited significantly reduced ovarian activity, less dominance behavior, and had lower dominance rank (Pandey and Bloch, 2021). Therefore, *Kr-h1* is associated with JH-mediated regulation of social organization in both bees, from labor division in honey bees to reproduction division and dominance rank in bumble bees. As a transcription factor, the target genes of *Kr-h1* involved in these processes need to beidentified in the future.

Recently, in the ant *Harpegnathos saltator*, *Kr-h1* has been demonstrated to regulate caste identity by acting as a core transcriptional regulator in response to the caste-specific JH and 20E signaling (Gospocic et al., 2021). *H. saltator* ants can switch between worker and queen-like status. When the queen dies or is removed from a colony, workers enter a dueling tournament until a few become reproductive individuals that are called gamergates (Sasaki et al., 2016). The gamergate abandons worker tasks such as hunting, begins to lay eggs, and exhibits dominant behaviors toward workers. This behavioral transition is accompanied by a reconfiguration of gene expression and cellular composition of the brain, and neurohormonal changes, such as the levels of JH3 and 20E (Penick et al., 2011; Sheng et al., 2020). By analyzing the transcriptional and behavioral response to JH3 and 20E *in vivo* and *in vitro*, the authors concluded that JH3 and 20E were responsible for the caste identities of workers and gamergates respectively and that *Kr-h1* was required to maintain the caste boundaries (Gospocic et al., 2021). ChIP-seq and RNA-seq analyses demonstrated that Kr-h1 functioned as a caste-specific transcriptional repressor by repressing “socially inappropriate” patterns of gene expression in the brain. In workers, *Kr-h1* is induced by JH3 and downregulates gamergate-biased genes. In gamergates, *Kr-h1* is downstream of 20E signaling and is involved in maintaining gamergate identity through the repression of worker-biased genes. Knockdown of *Kr-h1* in workers stimulated expression of gamergate-biased genes and inhibited hunting, whereas loss of *Kr-h1* in gamergates led to upregulation of worker-biased genes and promoted hunting (Gospocic et al., 2021).

### Maturation of Male Sexual Behavior

Besides regulating behavioral plasticity in females, *Kr-h1* is also involved in the maturation of male sexual behavior and male accessory glands (MAGs). In the noctuid moth, *Agrotis ipsilon*, newly emerged males are sexually immature and unable to behaviorally respond to the female-produced sex pheromone, and theirMAGs are undifferentiated and have a low protein content. Three to five days after emergence, males become sexually mature and highly attracted to the female sex pheromone; their MAGs exhibit elevated protein synthesis. This increase in behavioral responsiveness is well known to be JH-dependent (Duportets et al., 1998; Anton and Gadenne, 1999). Removal of the JH-producing CA induces a co-inhibition of the sex pheromone-triggered orientation flight and the MAG protein synthesis, which can be subsequently restored if operated males are injected with JH (Anton and Gadenne, 1999). The expression levels of *Kr-h1* and *Met* in *A. ipsilon* males are predominant in the antennal lobe and MAGs, wheretheir amount increased concomitantly with age, in parallel with sex pheromone responses and maturation of the MAGs (Duportets et al., 2012; Gassias et al., 2021). RNAi experiments show that males injected with either *Met* or *Kr-h1* dsRNA exhibited a significantly reduced MAG length and protein content (Gassias et al., 2021). Thus, *Kr-h1* and *Met* are critical for modulating JH-regulated male reproductive maturation.

### Embryogenesis

The initial studies on the role of *Kr-h1* in insect embryogenesis were conducted by Pecasse et al. (2000) and Beck et al. (2004) in *D. melanogaster*. Two deletion mutants (*Df(2L) Kr-h17.1* and *Kr-h1* ^*7id*^), obtained by the mobilization of the P element in one of the *Kr-h1* mutations (*Kr-h1* ^*7*^), that removed coding sequences common to both *DmKr-h1α* and *DmKr-h1β* rarely eclosed as larvae. However, the mutants showed normal embryonic development and elaborated larval structures by the end of embryogenesis, with occasional ectopic neurons (Pecasse et al., 2000; Beck et al., 2004). Later, in *B. mori*, Daimon et al. (2015) used the genome-editing tool TALENs (targeted mutagenesis mediated by transcription activator-like effector nucleases) to generate knockout silkworms with null mutations in *JHAMT* or *Met*. The transcriptional levels of *Kr-h1* were reduced dramatically in both mutants during the embryonic stage. Phenotypes examination found that the JH-deficient or JH signaling-deficient mutants showed high lethality in embryos and delayed embryonic development. Similar to the findings in *D. melanogaster*, embryogenesis appeared to be completed in the unhatched embryos, which were able to emerge as apparently normal first instar larvae if they were artificially dechorionated (Daimon et al., 2015). These results suggest that in the holometabolan, JH or JH signaling (JH-Met-Kr-h1) does not have a morphogenetic role in embryogenesis. On the contrary, studies by Fernandez-Nicolas and Belles (2017) in *B. germanica* showed that JH and its signaling have relevant functions during cockroach embryo development. Knockdown of *Kr-h1*, *JHAMT,* or *Met* by maternal RNAi technique not only caused impaired hatchability of embryos but also resulted in morphological malformations, such as failure formation of the germ-band anlage, imperfectly sealed dorsal closure, reduced abdomen, and intensely sclerotized cuticle (Fernandez-Nicolas and Belles, 2017). Therefore, involvement of JH and JH signaling in hatching appears to be conserved in hemimetabolans and holometabolans, whereas the morphogenetic functions were lost in holometabolan embryos. It is presumed that Br-C might be an important factor accounting for this distinction. In the *B. germanica* embryo, *Br-C* is consistently expressed during the period of JH production (Piulachs et al., 2010), its expression is reduced after JH or JH signaling is depleted (Fernandez-Nicolas and Belles, 2017). And the phenotypes observed in JH or *Kr-h1* depleted embryos of *B. germanica* are similar to those found in *Br-C* depleted embryos (Piulachs et al., 2010). On the contrary, in *B. mori* embryos, *Br-C* mRNA showed low levels during the cycle of JH production (Daimon et al., 2015). So it is supposed that the expression of *Br-C* would be induced prematurely in JH-deficient or JH signaling-deficient mutant *Bombyx* larvae. However, the *Br-C* expression levels remained very low in these mutations during embryonic development (Daimon et al., 2015). Even so, whether Br-C is the crucial factor determining the different functions of JH and JH signaling (JH-Met-Kr-h1) in embryogenesis between hemimetabolans and holometabolans remains to be investigated.

### Metabolic Homeostasis

Metabolic homeostasis plays important roles in insect development. *Kr-h1* has also been reported to control lipid metabolism. Studies in *D. melanogaster* found that *Kr-h1* mutations showed delayed larval development and reduced triglyceride (Kang et al., 2017). Under normal fed condition, *Kr-h1* acted on lipogenesis, whereas upon fasting, *Kr-h1* regulated lipolysis. Further, the authors demonstrated that insulin signaling was inhibited in *Kr-h1* mutant larvae and Kr-h1 could physically and genetically interact with FOXO to repress the transcriptional activity of FOXO on adipose lipase *brummer* (Kang et al., 2017). Previous studies by Mirth et al. (2014) showed that JH deficient *Drosophila* larvae pupariated at smaller size and FOXO is necessary for the size reduction. The co-regulation of lipid metabolism by Kr-h1 and FOXO contributes to a novel mechanism through which JH interacts with insulin signaling to integrate metabolism and growth during larval development.

## Conclusions and Prospects

Kr-h1 is a conserved transcription factor among insect orders. It acts as a cornestone regulator in insect life, from preventing metamorphosis to promoting female reproduction. In addition, it modulates social insects’ caste identity and behavior plasticity and the associated neuronal morphogenesis. Hence, *Kr-h1* could be a promising target in pest control in the future. However, despite the rapidly mounting evidence for the molecular and developmental functions of *Kr-h1*, many outstanding questions still need to be answered. The most pressing issues are: 1) What is the precise mechanism of Kr-h1 acting as both transcriptional repressor and activator? Is it dependent on the recruited cofactors? And what are these cofactors? So far, interactions of Kr-h1 with the coactivator CBP and the corepressor CtBP important for activating vitellogenesis and preventing metamorphosis have thus far been studied in *L. migratoria*. As *Kr-h1* plays many roles in insect life, we speculate that Kr-h1 interacts with multiple distinct partners, each time regulating a different aspect of insect biology. But how does Kr-h1 discriminate against these partners at different developmental stages? And how is their interaction stimulated? Moreover, besides phosphorylation, whether other post-translational modifications affect Kr-h1 binding with these cofactors? Does the opposite stage-specific regulatory effect of *Kr-h1* on *Br-C* depend on the property of partners or the post-translational modification of *Kr-h1*? In addition, Krüppel-like transcription factors can epigenetically induce DNA methylation or demethylation of the promoters of their target genes to inhibit or promote their transcription respectively. Does Kr-h1 exert similar functions? If so, what are the DNA methyltransferases and demethylase? 2) The functions of the eight zinc finger domains need to be dissected. Do all of them engage DNA? or which of them are involved in DNA binding and protein interaction? Do different zinc fingers recognize specific DNA sequences? 3) Apart from the already known functions, is Kr-h1 involved in other JH or 20E-regulated physiologies, such as spermatogenesis, testicular development, female mating, and sex pheromone production?In mammals, Krüppel-like transcription factors are critical in many physiological and pathological processes including cell proliferation, differentiation, inflammation, and apoptosis. Does Kr-h1 perform similar functions in insects? With the advent of new genomic technologies and the capacity of using different insect species, we will be in an excellent situation to deepen our understanding of the functions of *Kr-h1* and properly exploit it as the target of pest control.
